# Music as a social instrument: a brief historical and conceptual perspective

**DOI:** 10.3389/fcogn.2025.1533913

**Published:** 2025-02-24

**Authors:** Nicholas Bannan, Alan R. Harvey

**Affiliations:** ^1^Conservatorium of Music, The University of Western Australia, Perth, WA, Australia; ^2^School of Human Sciences, The University of Western Australia, Perth, WA, Australia; ^3^Perron Institute for Neurological and Translational Science, Perth, WA, Australia

**Keywords:** evolution, music, cooperation, sociality, reward, synchronization, dance

## Abstract

This article addresses the origins and purpose of communal music-making, including dance, and its role in human sociality. It accords special significance to the adapted nature of human vocalization, and the sensorimotor discrimination that allows the prediction and then generation of musically relevant, coordinated and simultaneous movements. Commencing with a historical survey of the development of ideas about the evolutionary importance of music in human social behavior, this mini-review then sets out to define and explore key issues involved in an evolutionary explanation. These include: acquisition and control of parameters required for vocal production (synchronization of pitch, timbre, duration and loudness); the exchange and transmission of pitched utterances in unison as well as in harmony; the roles of natural and sexual selection in shaping human musical abilities; the nature of cooperative behavior, and the consequences for social bonding of such interaction throughout life; transmission of such behaviors across generations, and the interaction between genes and culture that drives the evolution of complex social behavior in *Homo sapiens*. The article concludes with a brief review of current research that deals with contributory features of this field, especially in neuroscience which continues to provide important psychophysiological data that reinforces the long-held proposal that music has a key role in promoting cooperative, prosocial interactions leading to health and wellbeing over the human lifespan.

## Historical perspectives

Several recent publications concerned with the evolved, universal nature of human musicality have explored the relationship between the adaptive roles of music and social interaction and cooperation throughout life (e.g. Greenberg et al., 2021; MacDonald, 2021; Mehr et al., 2021; Savage et al., 2020, 2021; Leongómez et al., 2022; Stupacher et al., 2022; Wagner and Hoeschele, 2022). Each makes an important contribution to the field, yet speculation about the mystery of human musicality and its close links to human social behavior has a long history. This article sets out to summarize the traditions in philosophy, musicology and the social sciences that view the development of human musical behavior and associated collective expression as being inextricably linked to social culture. It goes on to summarize the influence of these perspectives on current research in anthropology, ethnomusicology and neurology that is contributing to building a more complete picture of the nature of human musicality and its role in social behavior and culture.

Rabinowitch (2020) provides a historical account of the capacity of music to effect social change, tracing this principally within the European philosophical tradition from the Ancient Greeks onwards. Plato attributed to specific musical phenomena (modes; instruments; Enea, 2022) the capacity for regulating moods and for establishing this response in childhood, with a moral purpose arising from the experience that distinguishes humans from animals (Bourgault, 2012). He also wrote that dancing makes humans more social: “For the whole life of man needs a good rhythm and a spirit of harmonious accommodation” [from *Protagoras*, quoted in Howes (1948)]. Chinese music theory from the 3rd century CE also attributed to musical stimuli the capacity to affect emotions (Kaufmann, 1976). The sixth century Roman philosopher Boethius attributed significance to musical quanta—rhythms and intervallic relationships—as affecting the relationship between the mind and the body (Enea, 2022). Within this tradition, Descartes (1649, cited in Jorgensen, 2012) enumerated the “passions of the soul” (wonder, love, hate, desire, joy, and sadness) that could influence and respond to music, a theory fleshed out in detail by the composer Mattheson (Mattheson and Harriss, 1739/1981) as the “doctrine of the affections” governing specific characteristics in musical form and expression.

Evolutionary insights into the role of music appeared prior to Darwin in the scientific speculation on music and language that arose during the French Enlightenment (Thomas, 1995). Rousseau (1763/2009), both philosopher and composer, considered music to be a species-specific trait, and explored how both music and language might have arisen. The Humboldt brothers, naturalist and explorer Alexander and philosopher-linguist Wilhelm provided reflections on the origins of vocal communication that proved influential for Darwin. Wilhelm wrote: “For man, as a species, is a singing creature, though the notes, in his case, are also coupled with thought” (von Humboldt, 1836/1999). Darwin's (1871) principal influence on the study of the origins of human vocalization is found in *The Descent of Man*. In his sexual selection theory, Darwin considered the musical nature of language origins and their social significance, the song-like origins of human vocalization important as females attracted mates and retained their affection (Piilonen, 2024). Spencer (1890) derived a theory of the origin of music through natural selection, but his model, which views music as an emotional variant of the existing capacity for speech, is in many ways the reverse of Darwin's.

A brief chronological presentation of publications since Darwin that reflect varied perspectives on the social nature of human musicality illustrates the accelerating recognition of this field as essential to an informed understanding of the human condition. Nietzsche accorded fundamental significance to music in human life, referencing evolutionary theory in his writing about the emotional basis of vocalization (Nietzsche, 1871), and the linguist Jespersen (1922) was a notable representative of his discipline in accepting a song-like origin for speech. Tylor (1871), the leading exponent of the new sub-discipline of cultural anthropology, drew on Darwin's own journal entries from the *Beagle* voyage as evidence of the role of music in social interaction in the South Sea Islands. Durkheim (1912/1995), who established the first Department of Sociology in Europe, assigned special significance to the role of ritual and its dependence on dance and music in achieving the state of social bonding that he termed “collective effervescence.” Indeed, the explorer Shackleton apparently chose people for his Trans-Antarctic expedition of 1914–1917 who could sing not only to facilitate coordinated work, but also because their ability to do so might well contribute to survival in the appalling conditions they would encounter (Philpott and Leane, 2016).

Seeking, on natural selection lines, to find features of music that are common to most or all musical cultures and identify the survival value that music lends, Roederer (1984) focused on three aspects: communication and attachment between mother and newborn (see also below); extraction of musical components of speech; and how music “equalizes the emotional states of groups of people.” In an important and influential book, Blacking (1976) also emphasized the vital role of music in defining, as well as separating, social groups. Storr (1992) explored his own passion for music in a wide-ranging historical and conceptual survey of sources illustrative of the significance of musical experience to human mental wellbeing, social interaction and spiritual conviction. Others have focussed on the roles of dance and physical drill as defining features of human social and collective engagement (McNeill, 1995), the importance of music in the achievement of altruism and cooperation (Ridley, 1996), and the vocal response to collective affiliation in the form of synchronous chorusing (Merker, 1999). More recently, Jordania (2022) has brought a new interpretation to bear on the synchronous chorusing model, suggesting a combination of predator-avoidance and male-bonding strategies that may have shaped group vocalization.

Freeman (2000) proposed how social bonding in response to such musical experiences is open to neurobiological explanation, while Brown (2000) proposed the term “musilanguage” as the precursor to a communicative split that saw speech and song arise from a common origin out of which they acquired independent characteristics. Mithen (2005) also depicted a form of musilanguage as the vocal basis for human enculturation, devising the term *Hmmmm* to capture its nature (holistic, multimodal, manipulative and musical) and attributing its acquisition to singing Neanderthals. In an important contribution, Levitin (2007) presented a synthesis of his and other researchers' work on how and why the brain has evolved to permit the wide range of operations that musical perception and production involve, discussing the likely role of human musical activity in enhancing social bonding and cooperation (see also Chanda and Levitin, 2013). Patel (2010, 2018) viewed music as a “transformative technology of the mind” (Patel, 2010) and in subsequent work suggested that the “invention of musical behavior triggered processes of gene-culture coevolution” (Patel, 2018).

Morley (2013) placed the latest developments in acoustic and psychological understanding within a chronological framework derived from archaeological and anthropological evidence that explored music's evolutionary origins. Dunbar (2012, 2023) also related archaeological and anthropological evidence to psychological investigations intended to reveal survival value. How might music increase pain tolerance, or promote bonding identification with the group? Tarr et al. (2015, 2017) investigated these issues through experimental work with dancers. Harvey's (2017) *Music, Evolution, and the Harmony of Souls* develops further the motivation of Storr (1992) to account for the peak experiences and collective cognitive and emotional states that music can evoke, emphasizing a key role of communal music-related activities in enhancing trust, empathy and altruism. Such interactions require an ability to recognize and understand the likely mental state and intentions of others (Theory of Mind), a social-cognitive task that may have been closely linked to the emergence of music (Livingstone and Thompson, 2009; Harvey, 2018).

A fruitful complementary field of study regarding the purpose and development of human vocalization arose in the study of musical response from before birth onwards (Roederer, 1984; Woodward, 2019), and especially the vocal, tactile and gestural interaction of mothers and infants. Kessen et al. (1979) analyzed the matching of pitch to their mother's singing of sustained tones in the voices of infants aged between 3 and 6 months as a window that closes as the child begins to explore vocal production more widely and individually. Trevarthen (1979) similarly examined the vocalizations of infants as contributory to the eventual acquisition of speech. Trainor and Trehub (1993) considered the listening strategies of infants as foundational to music as well as speech development. Dissanayake (2008) considered the survival advantages of the behaviors involved in such mother-infant interactions, and their subsequent adult role in reproductive success, suggesting that they form a link to lifelong expression of affection that places multimodal sensory exchanges at the heart of the human capacity for intimacy and its role in trust and mutual affection.

## Evolutionary theory and human social behavior

As part of this brief review into music as a social instrument, it is pertinent to comment on the origin of complex and rewarding prosocial interactions in humans. Perhaps the most widespread phenomenon that plays a part in group synchronization is collective movement. Inter-individual synchronization of movement, and sometimes vocalization, is not unique to *Homo sapiens* and has been reported in other species (e.g. Ravignani and Norton, 2017; Ravignani, 2019; Bouwer et al., 2021; Seki, 2021), but there may be specific adaptations to auditory-motor circuitry that have evolved in humans (Patel, 2008). Stewart (1998) presented mathematically-derived models for how coordinated movement is achieved, from which one might conclude that, while marching and dancing may be seen as equivalent in humans to the shoaling of fish and the flocking of birds, the complex properties of group singing and dancing that we will discuss represent a further and more elaborate correspondence. Indeed, recent genetic studies provide evidence for inheritance of, and even positive selection for, some elements of inter-individual synchronization, music-related ability and creativity (Liu et al., 2016; Oikkonen et al., 2016a,b; Mariath et al., 2017; Beccacece et al., 2021; Niarchou et al., 2022; Yeom et al., 2022). Furthermore, a few genes related to beat synchronization are located in so-called human accelerated regions (HARs). HARs are human-specific and affect genes involved in, for example, neural development and expansion of some cortical areas (Guardiola-Ripoll and Fatjó-Vilas, 2023). Of the almost seventy gene loci associated with rhythm perception and beat synchronization (many enriched in fetal and adult brain tissue), two were reported to contribute 2.26 times more to observed heritability than would be expected if there was a uniform distribution of variants (Niarchou et al., 2022).

Stewart's (1998) analysis of the coordinated locomotion of widely different species in the media of water, air and on land observed the patterns of limb-movement that give rise to the multi-sensory exchange on which synchrony depends. A parallel investigation by Hockett (1960) focussed on the design features of acoustic communication in a representative range of species from which the specific requirements for the human capacity for language could be assembled. His model illustrates that, while significant individual aspects of human communication are shared with many other species, both through convergent and divergent evolution, human speech is the only example of a communicative ability that ticks every box. While song and speech overlap in relation to Hockett's criteria, the element that lends itself most both to the link between the two and the distinct achievement in vocal perception and production of our species is our adaptive aural response to harmonicity (Bannan et al., 2024a,b).

Singing in harmony is rare in other species, but universal and essential in humans if fathers are to sing in unison with female partners and children. More elaborately, the vowel system on which all languages depend, albeit significantly diverse in quantity and kind (Ladefoged and Maddieson, 1990), employs timbral variants of the fundamental pitch produced by the larynx, termed source-filter production (Fant, 1970). Nikolsky (2020), comparing the relationship between formant frequencies in language and musical timbres in song and jaw-harp performance, concluded that they have influenced one another in their dependence on response to the harmonic series. Bannan (2022) commenced a systematic review that included analysis of the relationship between the perception of harmony in music and the distinct timbral quality of vowel sounds. The critical aspect of human vocalization that permits both speech and song demands investigation in all its dimensions: acoustic, anatomical, semiotic and psychological.

Melis (2018) considered comparative studies with closely related primate species to be crucial to understanding the origins of human prosociality, focusing particularly on the developing behavioral responses of human and chimpanzee infants. Collaborative behavior emergent from “underlying proximate mechanisms” (Duguid and Melis, 2020) can be characterized in four sequenced categories that can be observed in primates: by-product collaboration (in which individuals respond similarly to the same stimulus); socially influenced collaboration (individuals can be influenced by observation of others in their response); actively coordinated collaboration (a learnt collective response arising from previous experience, possibly arising from a bonded relationship); and collaboration based on shared intentionality (O'Madagain and Tomasello, 2022). This sequence could plausibly be applied to how the development of vocalization related to human social and cultural coordination. Marching in-step remains a universal expression of shared physical commitment (McNeill, 1995), the synchrony of the lower limbs leaving the arms free to carry (especially in transporting infants yet unable to walk) or wield tools and weapons, providing the metric underpinning for vocal synchrony and chorusing, suggested to be a distinct stage in the development of vocal communication prior to language (Merker, 1999, see also Patel, 2024).

We have already briefly mentioned suggested links between music and Theory of Mind (Livingstone and Thompson, 2009; see also Harvey, 2017). In humans, trust and reciprocal altruistic behavior between non-relatives can be beneficial to individuals but can also prove favorable to the group as a whole (Trivers, 1971; Kurzban et al., 2015; Silk and House, 2016), facilitating the sharing of information and diverse physical resources. In humans, the proposed evolutionary mechanism of group selection (Hamilton, 1964a,b) is associated with inclusive fitness and greater social and cultural adaptability (Richerson et al., 2016), a condition in which “natural selection favored genes that gave rise to new, more pro-social motives” (Boyd and Richerson, 2009). There remains some controversy about cultural group selection theory [see for example commentaries in Boyd and Richerson (2009)], although evidence strongly supports the importance of cooperation, norm-abiding behavior and positive feedback in promoting emotional alignment and social interaction (Korn et al., 2012; Tomasello and Vaish, 2013; Prehn et al., 2015). “Humans are the only multilevel species in which mere tolerance between interbreeding groups evolved into multigroup cooperative networks and the coordination of whole social groups, giving rise to many derived features of human sociality such as intense cooperation, prosociality, and cultural transmission” (Greuter et al., 2012). Of course, altruism can sometimes prove costly to an individual, but in times of stress or conflict, interactions that promote reliable collective action (e.g. hunting, food sharing, fighting) likely improve the survival potential of group members and their kin (Bowles, 2006). The potential impact that musical activities can have in such circumstances is discussed further below.

## Musicality and social interaction

In humans, and in contrast to the music universal, language is the cognitive vehicle used to express thoughts and creative ideas, it is the primary means of communicating knowledge and learned skills within and between generations. It contains past, present and future tenses that allow “mental time travel” and a shared appreciation of the past, present and future. So why music? Why does this expensive endeavor in time and energy remain a universal, why does it remain “a permanent part of our mental furniture” (Storr, 1992)? Paraphrasing this author, music is not usually propositional, does not present testable hypotheses or set down precepts. Several proposals have been put forward that almost certainly have common foundations, at least at the neurological level. In addition to the Darwinian idea of courtship and mate selection, music has also been suggested to derive from the need to develop parent-infant attachment before babies learn to talk and use language (DeCasper and Fifer, 1980; Trehub, 2000; Falk, 2004; Levitin, 2007; Dissanayake, 2008; Trevarthen, 2002). This early communication, important in driving the behavioral, perceptual, cognitive, emotional and social development of preverbal infants, is generally known as motherese, and already employs the meaningful production and perception of variation in the four parameters of pitch, timbre, amplitude and duration that represent musical thinking and feeling (Bannan, 2008). Lastly, and the primary subject of this brief review: in childhood, adolescence and beyond, music yokes physiological arousal, stimulates cooperative group behavior and survival strategies, enhances trust and prosocial interactions, and helps to define social identity. In essence, it “intersects with cultural boundaries, facilitating our ‘social self' by linking our shared experiences and intentions” (Schulkin and Raglan, 2014).

Dissanayke's persuasive model (2008) for the relationship between musical interaction and sexual selection provides the basis for tracing a timeline that represents the nature of its potential effect over the human life cycle. Behaviors enacted initially in the mother-infant dyad to provide security and affection (touching, hugging, kissing, imitative facial expression, vocal duetting: see also Trevarthen, 1979, 2002) reappear as the multi-sensory repertoire through which adults convey and sustain affection. This sexual selection role that Darwin (1871) identified as the basis of human vocal communication has informed several recent experimental studies, including Marin and Gingras (2024), Bamford et al. (2024), and Keller et al. (2023), that illustrate how music can affect sexual attraction both in shared listening and in performance. In even later life, these behaviors remain available as means by which continued active participation in singing and dancing, as well as nostalgia arising from musical memories, can contribute to prolonged emotional wellbeing.

Between the mother-infant dyad and the adult romantic phase there is the development of peer-interactive musical play behaviors (Romet, 1980; Kartomi, 1999; Opie, 1993). When studying children in Western Java, Romet (1992) found that, over time, infants cease to engage in the pattern of behavior represented by interaction with their mothers and prefer the new enticement of songs and movement games learnt from their peers. The rebellious nature of this departure represents a new context for creativity, built upon during later childhood and adolescence, that has a vital role in the transmission of culture and the social engagement in play that prepares for adult activities. Of course, while musical participation often leads to positive outcomes—therapeutic, informative, happy, inspiring, enriching—music, rather than being neutral or beneficial, is sometimes seen as a threat and can be exploited and used with malicious and unpleasant intent. As Jordania (2022) argues, it can prepare for conflict and lower pain thresholds. But as much as these may aid predator avoidance and induce the mindset on which successful hunting depends, they may also be deployed in warfare (see also Bowles, 2006). One nation's stirring anthem may be the death knell of its victims, while music has on occasion been employed as a means of torture (Peters, 2019). That the power of music is open to such ill-use is an inevitable consequence of its shared adaptive history with the complex behavioral repertoire of our species.

## Music as a driver of social behavior—Recent neuroscience findings

Building on the historical philosophical, anthropological and ethnomusicological perspectives that view the development of human musical behavior as being inextricably linked to social culture, to what extent has modern psychophysiological research reinforced this position? Is there a neurobiological basis that helps understand how, and perhaps clues as to why, humans continue to maintain two related yet distinct universal communication systems—language/speech and music? Bannan (2022) addressed this issue as representing “two servants, one master,” discussing the similarities in acoustic processing of these two communication streams. There are indeed common components of auditory analysis such as the processing of pitch, timbre and rhythmicity (Rogalsky et al., 2011; Perrachione et al., 2013), and while the distinction between music and language is not always as clearcut in all cultures (Cross, 2021; Ozaki et al., 2024), there are differences in left vs. right hemisphere functionality (Albouy et al., 2020). Recent functional imaging has identified separate regions in cerebral cortex that process only music, or only language (Angulo-Perkins et al., 2014; Norman-Haignere et al., 2015; see also Sihvonen et al., 2024). There is even a report, based on intracranial recording and functional imaging, of a neuronal population in human auditory cortex that is selective for song (Norman-Haignere et al., 2022).

As sophisticated imaging equipment for investigating brain function has become increasingly available, further insights into the neural organization of complex musical processing in humans have been revealed. Peretz and Zatorre (2005) were among the first to systematically review research on the workings of aural perception, as well as the processing of not only pitch and timing, but also musical memory and diverse emotional responses. They also discussed early neuropsychological research on singing, playing an instrument, reading music notation, and the effects of musical training on neural networks. Beyond primary and secondary auditory cortex in the superior temporal gyrus, numerous “higher level” cortical areas have been found to be activated by listening to music that elicits strong emotional responses (either positive or negative). These include several regions within prefrontal cortex, inferior frontal cortex and the supplementary motor area. Singing or playing music obviously involves activity in other regions including motor cortex, cerebellum and parts of the basal ganglia. In addition, music also activates components of the limbic system (hippocampus, parahippocampal gyrus, amygdala and cingulate cortex), critically important in a number of functions including learning, memory, motivation and emotional responsiveness. Music is often arousing and rewarding, and induces activation in the nucleus accumbens in ventral striatum, an anticipatory and reward center (e.g. Blood and Zatorre, 2001; Chanda and Levitin, 2013; Zatorre and Salimpoor, 2013; Ferreri et al., 2019). Furthermore, the social sharing of musical experiences is especially rewarding and enhances pleasure (and memory of the event) even further (Curzel et al., 2024).

Communal music-making and the increased empathy and social bonding that results (Kirschner and Tomasello, 2010; Rabinowitch et al., 2013; Pearce et al., 2015; Savage et al., 2020), requires sensorimotor matching of vocal parameters such as pitch, loudness, timing and duration (Bannan et al., 2024b). It is also expressed multi-modally through the tendency of humans “to temporally coordinate when engaging with each other” (Hoehl et al., 2021), relating vocal production also to gesture and facial expression as well as gait. Synchronized chorusing is the prime sensorimotor component of the specific human capacity for communal music-making, requiring the mutual recognition and production of pitch in unison and in harmony and operating within the parameters of rhythm, including meter and tempo, as well as pitch. This complex group behavior requires sophisticated sensorimotor processing, involving coordination between breathing, vocal cords in the larynx, and muscles controlling the pharynx, jaw, lips, tongue and so on. In this regard it may be relevant that in humans there are specific projections from motor cortex to the motor neurons in the brainstem that control vocalization (Zarate, 2013) and enhanced connectivity between regions interconnecting laryngeal motor cortex with sensory processing regions, presumably increasing sensorimotor integration (Kumar et al., 2016).

Social collaboration is greater during music improvisation (Keeler et al., 2015; Walton et al., 2018; Hadar and Rabinowitch, 2023) and silent-disco dancing in time together enhances social memory of others in the group, likely involving gaze and enhanced visual attention (Woolhouse et al., 2016; Behrens et al., 2020) although under some conditions facial cues may not be necessary for developing synchronous interactions in young children (Rabinowitch et al., 2024). Links between sound and movement are well-established in many species; in human imaging studies, music and movement are closely linked (Sievers et al., 2013; Karpati et al., 2016; Laland et al., 2016; Levitin et al., 2018), and the term “groove” has been coined to describe the pleasure derived from movement in time to music (e.g. Witek et al., 2014; Matthews et al., 2020). Successful coordination in a group context requires real-time adjustments and is also associated with activation of reward centers within the brain (Menon and Levitin, 2005; Salimpoor et al., 2011; Koelsch, 2018). Prediction of action is also needed, and it has been reported that there is increased activity in premotor cortex, basal ganglia and cerebellum when silently anticipating a known musical sequence (Leaver et al., 2009). Neural entrainment (the temporal relationship between an external stimulus and an internal rhythmic response) to music in individuals is a well-established phenomenon (Trost et al., 2014). This is clearly an important attribute when coordinating interactive, presumably prosocial behaviors. Brain networks associated with interpersonal coordination of rhythmic behavior have been identified (Harry et al., 2023), and it is also of interest that recent research has found there is synchronization of brain wave activity (Schirmer et al., 2016; Müller et al., 2021; Cheng et al., 2024) in groups playing musical instruments (Shahal et al., 2020), when singing in choirs (Delius and Müller, 2023), and when moving/dancing to a meter or beat in coordinated groups (Dalla Bella et al., 2015; Hu et al., 2022). Such synchronization and “brain-to-brain coupling” may help promote interpersonal coordination (Hu et al., 2022; Delius and Müller, 2023).

Finally, with regard to what is known about the neurochemistry of music-induced prosocial behaviors, functional imaging studies have revealed similarities in the cortical regions that are active when performing non-selfish tasks requiring mutual cooperation (Rilling et al., 2002), and when listening to music that is familiar and rewarding (Harvey, 2017, 2018). While co-activation of brain regions does not necessarily indicate shared neural circuitry (Peretz et al., 2015), these regions also overlap with areas involved in oxytoninergic processing and contain oxytocin receptors (Harvey, 2020; Arnold et al., 2024). Both oxytocin and endorphin have been linked to musical activities, including dance (Dunbar, 2012; Kreutz, 2014; Keeler et al., 2015; Tarr et al., 2015, 2017; Yuhi et al., 2017; Good and Russo, 2022), and both—along with dopamine—influence important aspects of human sociality (Chanda and Levitin, 2013; Baskaran et al., 2017; Pearce et al., 2017, 2018; Harvey, 2020). These neuromodulators are also critically important in maternal-infant attachment behavior (Izaki et al., 2024), reinforcing the impact they have across the human life-cycle and their continued role in human social behaviors, with music a major driver. Note that the exact contribution of these neuromodulators to the impact that musical activities have on human sociality may require further study because there are potentially complex interactions of all three of these systems at the receptor and synaptic level (Arnold et al., 2024; Izaki et al., 2024; Borland, 2025; Yao and Kendrick, 2025).

## Conclusions

We began by citing recent, influential publications that have emphasized key links between the evolution of human musicality and prosocial behaviors. Here we remind readers that the publication of ideas relating to the nexus between music and prosociality has a long history in the scholarly fields of philosophy, musicology and social science, and emphasize how modern research and new technologies have only served to reinforce and interpret this view. But this is not merely an academic exercise since in the 21st century the impact of music on human sociality, empathy and overall wellbeing across the lifespan remains just as important as it was for our ancestors. Music has beneficial effects on general health and a range of cognitive functions, and its value in therapy is increasingly accepted (e.g. Brancatisano et al., 2020). Its use in aiding in the treatment of age-related motor (e.g. Fan et al., 2023; Huang et al., 2024) and memory disorders (e.g. Bannan and Montgomery-Smith, 2008; Jiménez-Palomares et al., 2024; Sun et al., 2024) is well-known, as its use in reducing the experience of pain (Arnold et al., 2024). At the level of brain circuitry, musical expertise and music-making including choral singing consistently enhances the structural integrity (and it is assumed functionality) of some white matter tracts (Leipold et al., 2021), even in aged populations (Moisseinen et al., 2024). But in continuing to improve our understanding as to the psychophysiological basis that links musical activity with prosociality we will, to suggest just a few examples: (i) significantly improve social engagement in young people with autism spectrum disorders (Shi et al., 2024); (ii) further improve the efficacy of music therapy in awakening positive, social interactions between persons with dementia and their caregivers (where language may already have been impaired) or in choirs (Ridder et al., 2024); (iii) facilitate relationship completion and improved quality of life for patients in palliative care *and* their families (Ramesh, 2024). Such health benefits as these therapeutic procedures may achieve clearly build upon the specific evolved characteristics that shaped our species in prehistoric times.
